# A case report of detecting subclinical coagulopathy in a patient with boomslang (*Dipholidus typus*) bite

**DOI:** 10.4102/safp.v63i1.5299

**Published:** 2021-08-11

**Authors:** Mungela J. Tambwe, Vidya Lalloo, Andreas Engelbrecht, Pholosho Pelle

**Affiliations:** 1 Department of Family Medicine, Faculty of Health Sciences, University of Pretoria, Pretoria, South Africa

**Keywords:** boomslang bite, monovalent antivenom, haemotoxic envenomation, snake bite, rotational, thromboelastometry (ROTEM)

## Abstract

The boomslang (*Dipholidus typus*) has a predominantly haemotoxic venom. Because of the consumptive nature of the coagulopathy, signs and symptoms are usually delayed by up to 72 h after the bite. Traditional laboratory coagulation assays have a long turnaround time, by which time the patient’s bleeding and clotting profile has changed. A 25-year-old male patient was bitten by a boomslang. Despite two normal laboratory coagulation assay results, a point-of-care rotational thromboelastometry showed low fibrinogen levels, leading to the administration of monovalent antivenom. This report highlights the value of point-of-care thromboelastometry in the care of patients with subclinical boomslang envenomation.

## Introduction

The boomslang is a medium-sized tree-dwelling snake with an oval head and characteristically large eyes. Male snakes are typically green but may be two-toned in bright yellow and black. Female snakes tend to be brown in colour.^1^ Boomslang bites usually occur from direct handling of the snake and lead to morbidity and mortality from a venom-induced consumptive coagulopathy (VICC).^2^ Signs and symptoms may appear within 4 h but may be delayed for as long as 72 h.^3^ Coagulopathy occurs primarily from the activation and consumption of factors II, IX and X.^2^

Bleeding may range from minor bleeds at punctures sites, epistaxis and bruising to life-threatening bleeding into muscles leading to haemoglobinuria and acute tubular necrosis, gastrointestinal bleeding and central nervous system bleeding.^4^ Life-threatening disseminated intravascular coagulopathy (DIC) is the outcome if antivenom is not administered.^5^ Bedside investigations such as 20-min clotting time are useful to establish an impression of coagulation. Laboratory investigations including prothrombin time (PT), activated partial thromboplastin time (aPTT) and international normalised ratio (INR) have traditionally been requested to detect coagulopathy and provide baseline values to monitor the patient’s clotting profile. The use of thromboelastography (TEG) and rotational thromboelastometry (ROTEM) has become a standard in many trauma units and can be useful to support patients with boomslang envenomation with specific blood products.^6^

Antivenom is the definitive treatment for boomslang bites. South African Vaccine Producers (SAVPs) produces a boomslang-specific monovalent antivenom (MAV). One to two vials of MAV are usually sufficient to neutralise the venom from a boomslang bite, but administration of additional vials may become necessary if the patient is still coagulopathic 2 h – 4 h after the initial dose. If administered early, coagulopathy may be avoided completely. As the SAVP MAV is produced from equine serum, it contains proteins that have allergenic potential. If anaphylaxis occurs, immediate administration of intramuscular adrenaline is mandatory in a dose of 0.5 mg. Milder forms of allergic reactions can be treated with antihistamines and steroids.^3^

## Case report

**FIGURE 1:**
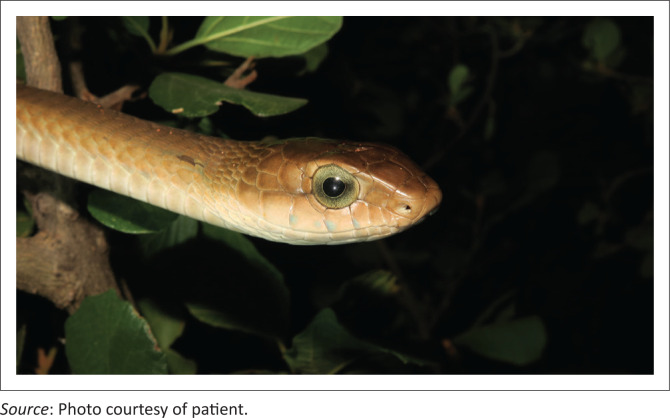
Culprit boomslang: A 1.2 m adult female.

A 25-year-old male patient, Mr S, presented to the emergency centre (EC) after being bitten by a boomslang. He is an otherwise healthy herpetologist who was employed in Limpopo province, South-Africa, to conduct a herpetofauna survey for a game reserve. He was accidentally bitten by a boomslang which he was handling during photography (Figure 1). A single fang of the snake reportedly nicked him on his hand.

Mr S was bitten at 11:35. He was initially taken to a local hospital in Polokwane. Because of the unavailability of MAV at the hospital, the patient was transferred to Steve Biko Academic Hospital (SBAH).

**FIGURE 2:**
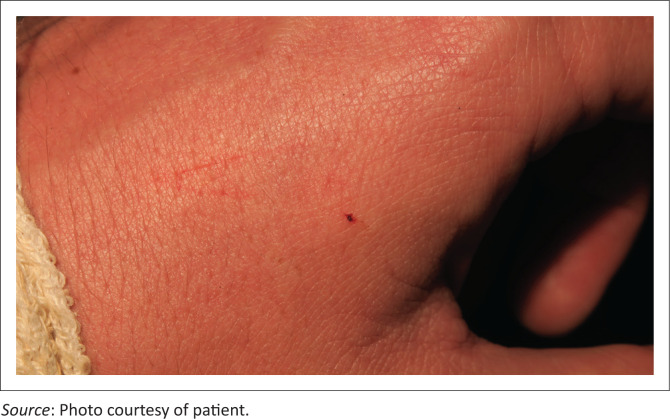
Trivial wound with a single fang puncture mark and superficial scratch.

It took 9 h from the time of the bite, to Mr S’s arrival at the SBAH EC. Mr S walked unaided into the EC, and reported no symptoms. His vital signs were within normal limits. Physical examination revealed a small fang mark at the base of his left index finger with a 1-cm superficial scratch medial to the puncture site (Figure 2). The superficial scratch was believed to correlate with the row of maxillary teeth. The typical appearance of a ‘true bite’ is that of two deep fang punctures. There was no bleeding at the bite site. The rest of the physical examination was normal. The appearance of the wound together with the lack of symptoms led to the consideration of a ‘dry bite’ (a bite without envenomation). An intravenous line was inserted in the right antecubital fossa, and caused no oozing. A coagulation assay was requested, including an INR, PT and PTT. Urea, electrolytes and liver function tests were also ordered and within normal limits.

A whole blood clotting test was performed which clotted within 5 min. A detailed history of the bite revealed that the snake’s one fang nicked the patient. The fangs did not fully penetrate the skin and the snake did not ‘chew’ or hang on for a period of time allowing the venom time to be injected. This history, together with only the single fang mark, the lack of symptoms 9 h post bite, the 5 min clotting time and the reassuring coagulation assay results from the hospital in Polokwane, led to the decision to observe the patient overnight. Tetanus toxoid 0.5 mL was administered intramuscularly and an ROTEM was scheduled for the following morning at 7:00.

**FIGURE 3:**
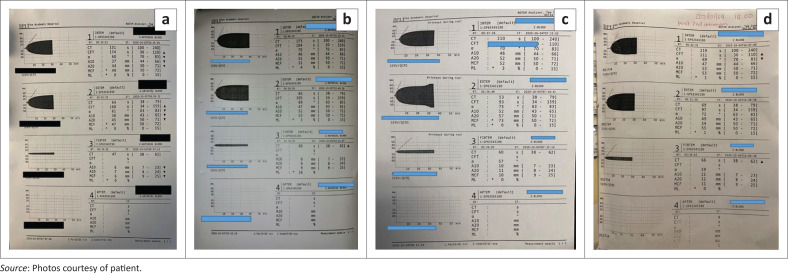
Rotational thromboelastometry. (a) Day 1 post bite showing abnormalities in the intrinsic and extrinsic pathways, as well as low fibrinogen levels. (b) Done at 16:00 on day 1, showing marked improvement after antivenom administration. (c) Essentially normal rotational thromboelastometry on day 2, but with the fibtem values on the lower end of the normal range. (d) Six hours after the second vial of monovalent antivenom was infused on day 3.

Day 1 post bite, the patient remained subclinical. His coagulation assay obtained that the previous night was normal. The ROTEM, however, revealed abnormalities on the intem, extem and fibtem analysers indicating abnormalities on the intrinsic and extrinsic clotting pathways, as well as low fibrinogen levels. (see Figure 3a.)

**FIGURE 4:**
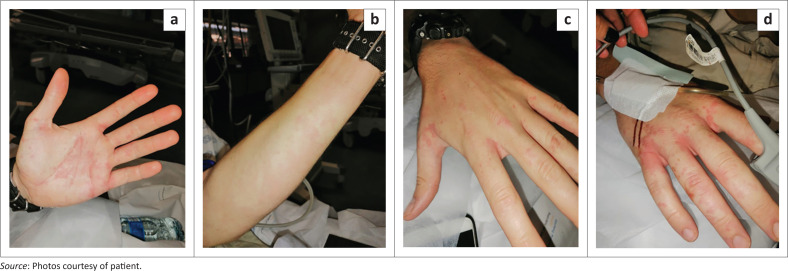
Allergic reaction after antivenom administration. (a–d) Uriticaria expression of allergic reaction.

The patient was being nursed in a monitored bed. Based on the ROTEM result, the decision was made to administer 1 vial of SAVP MAV in 50 mL normal saline as a slow infusion over 30 min. Premedication with adrenaline 0.25 mg subcutaneously was administered. Twenty minutes into the infusion, Mr S developed a generalised urticaria (see Figure 4). His chest felt heavy and his throat felt ‘scratchy’. His lung sounds remained clear with no wheezing and his blood pressure and saturations were maintained. The antivenom infusion was stopped immediately. Promethazine 25 mg and hydrocortisone 100 mg were administered intravenously. The patient responded well to these measures and the antivenom infusion was restarted 2 h later at half the rate.

The repeat ROTEM 9 h later (Figure 3b) showed a markedly improved intem and extem with only a clotting time abnormality persisting on the fibtem. Five units of Cryoprecipitate were administered.

On day 2, the patient was still asymptomatic. A 20-min-clotting-time test was performed at 07:00 and although a partial clot was formed by 20 min, the entire blood specimen was not clotted by the time an hour had passed. This prompted a third ROTEM (Figure 3c). Although the ROTEM was technically normal, the fibtem levels were all on the low range of normal. This together with the failed 20-min-clotting-time test prompted discussion about the risks of subsequent anaphylaxis and the additional cost of another vial of MAV versus the benefit of preventing clinical VICC, the cost of blood products (financial and transfusion reactions) and preventing a prolonged hospital stay. The decision was taken to go ahead and administer a second vial of MAV. This was performed under controlled circumstances with intubation equipment, a ventilator and adrenaline on standby. Adrenaline pre-treatment was administered. The antivenom infusion although mixed in the same manner was administered over a longer period of time (40 min). As the infusion was near completion, Mr S again developed a generalised urticarial skin reaction and scratchy throat, which settled with promethazine and hydrocortisone. This time he did not experience any chest tightness. His vital signs remained normal. Based on the delayed bleeding from boomslang venom (up to 72 h), the patient was kept for further observation for another day.

On day 3, the patient remained subclinical. A repeat ROTEM (Figure 3d) showed minor abnormalities. A repeat whole blood clotting test clotted in 6 min and the patient was discharged with instructions to return if he developed any symptoms. In spite of a slightly deranged Viscoelastic assays (VEA), full clinical recovery was anticipated because of the administration of the second dose of MAV and blood products. The patient was advised to return if clinical VICC developed.

## Discussion

This case demonstrates that ‘minor’ boomslang bites can lead to a VICC. Boomslang venom contains several proteins responsible for the coagulopathic effects it has on blood, including phospholipase A2, snake-venom serine proteinases and snake-venom metalloproteinases that lead to the consumptive coagulopathy.^7^ Venom-induced consumptive coagulopathy starts with prothrombin activation (IIIa) and activation of factors IX and X.^2^ This leads to fibrinogen being converted to fibrin, reducing circulating fibrinogen levels. As time passes, more factors are consumed. Platelet consumption with thrombocytopenia is common after envenomation. If left unchecked, DIC ensues leading to death.^7^ A number of case studies^4,8^ have shown derangements of whole blood clotting time and standard coagulation tests (PT, PTT, INR and fibrinogen); however, there have been no direct comparisons of coagulation tests with VEA in the setting of boomslang bites. This emphasises the importance of this case report. It is well documented that aggressive supportive measures may not save the life of a patient when antivenom is unavailable.^9^ Once a patient deteriorates to clinical VICC, the consequences are life-threatening and costly.^4^

The use of objective real-time whole blood coagulation studies like ROTEM/TEG can be helpful in the early diagnosis of life-threatening bites. They may indicate the need for the early use of antivenom leading to an uncomplicated recovery.^7^ Antivenom has been shown to be effective in case studies.^2,8^ Rotational thromboelastometry or TEG is also useful to help choose the most appropriate blood products and treatments necessary for supportive management of coagulopathic snakebites.^10^

Our case highlights the utility of ROTEM to diagnose envenomation before symptoms in the case of a suspected dry bite.

## Conclusion

This case study highlights the utility of ROTEM to identify evidence of envenomation by a haemotoxic snake in the context where the history and clinical findings are not convincing of envenomation. Rotational thromboelastometry was also useful to aid in the choice of therapy of the VICC. Unfortunately, VEA is not widely available in developing countries. Until this test becomes more accessible, strategies to improve outcomes may include observations for clinical signs of VICC for at least 72 h, repeat conventional coagulation assays 12 hourly and repeat the whole blood clotting time test 8 hourly.

## Dissemination of results

This case was discussed on an academic ward round with SBAH emergency medicine doctors led by the co-authors, Dr Vidya Lalloo and Prof. A Engelbrecht. The case was also discussed at the monthly academic meeting with emergency medicine faculty. We plan to share the link to the published article on various platforms such as our hospital WhatsApp groups and on the Emergency Medicine Pretoria social media platforms (Twitter, Facebook).

## Appendix 1

**TABLE 1-A1:** Sequence of events from the snake bite to the time patient was discharged from the hospital.

Time	Event
11:35	Bite
20:30	Arrival at Steve Biko Hospital
20:40	Coagulation panel, whole blood clotting test
Day 1	ROTEM: Abnormal administration of Antivenon, Urticaria
Day 2	20 min clotting time abnormal ROTEM: Abnormal administration of 2nd Antivenon Urticaria
Day 3	Patient subclinical discharge from the hospital. ROTEM: Minor abnormality
End of week 1	Telephonic contact, patient asymptomatic.

ROTEM, rotational thromboelastometry.
